# Management of Long Bone Fractures in Two Dogs Using 4.5mm Dynamic Compression Plates

**DOI:** 10.1155/crve/6524481

**Published:** 2025-09-15

**Authors:** Jessica Hynes, Helena Rylander, Peter Muir

**Affiliations:** ^1^Comparative Orthopaedic Research Laboratory, Department of Surgical Sciences, School of Veterinary Medicine, University of Wisconsin-Madison, Madison, Wisconsin, USA; ^2^Department of Medical Sciences, School of Veterinary Medicine, University of Wisconsin-Madison, Madison, Wisconsin, USA

**Keywords:** dog, 4.5mm bone plate, fracture, humerus, radius and ulna

## Abstract

The objective of this study was to describe clinical outcomes associated with the use of the 4.5mm dynamic compression plate (DCP) for repair of long bone fractures in two giant breed dogs. Case 1, a 6-year-old male Great Dane, underwent surgical stabilization of a comminuted antebrachial fracture with a 4.5mm broad DCP fixation of the radius in addition to a 3.5mm seven-hole locking compression plate (LCP) that was placed on the ulna. The patient developed a recurrent surgical site infection that was considered a major complication. Follow-up 2 years later demonstrated clinical union of the fracture radiographically, with a Grade 1 of 5 lameness of the thoracic limb and limited range of motion in the carpus. Case 2, a 1-year-old male Saint Bernard, underwent surgical stabilization of a short oblique humeral fracture with a 4.5mm broad DCP fixation. The patient experienced a delayed union and required revision surgery for bone graft placement 3 months after surgery. Clinical union occurred 8 months postoperatively. The use of 4.5mm DCP for long bone fracture repair in two giant breed dogs was associated with successful fracture healing and an acceptable outcome without implant breakage in the face of delayed bone healing that was associated with major complications and continued lameness.

## 1. Introduction

Long bone fractures are one of the most common orthopaedic problems in veterinary medicine. Around 75% of long bone fractures are caused by trauma, primarily secondary to vehicular accidents [1]. Although there are numerous treatments for fractures, the most common general recommendation is open reduction and internal fixation (ORIF) to ensure return to normal function of the limb with the lowest risk of complications [1].

The primary goal of surgical treatment is to create a fracture construct that can sufficiently resist cyclic loading and enable fracture healing clinically. As veterinary patients require human supervision to limit construct loading during the healing period, implant selection is primarily based on this goal. In general, plate constructs and interlocking nails are the preferred treatment for long bone fractures due to their ability to resist the multiaxial mechanical forces placed on the bone [1]. Large or giant breed dogs require a stronger construct to resist the increased forces that will be applied to the fracture construct during the healing period due to their increased size when compared to smaller dogs [2–4].

Implant failure occurs either because of biologic failure of bone healing or mechanical failure of the fracture–implant construct [2, 3]. Mechanical failure can be caused by acute single event overload of the implant. However, it is most often the result of cyclic loading leading to fatigue failure, which can occur at any point of the healing process [4]. The most common mode of implant failure is related to bone screws either loosening or breaking, likely due to their strength when compared to bending or breakage of the plate itself, which has a higher area moment of inertia (AMI) than the screws [3, 4]. Therefore, use of a larger plate and screw size in large or giant breed dogs should help prevent failure due to cyclic loading. Appropriate use of a larger plate and screw size should follow standard orthopaedic guidelines relative to bone size [5].

Currently, there is limited data on the clinical use of plates and screws larger than the 3.5mm broad dynamic compression plates (DCPs), limited contact dynamic compression plate (LC-DCP), or locking compression plates (LCP) for fracture repair in dogs. Therefore, the aim of this case report was to describe clinical outcomes with the use of the 4.5mm DCP plates and screws for the repair of long bone fractures in two giant breed dogs. In this regard, the use of plates combined with 4.5mm screws appears particularly helpful in cases with delayed healing.

## 2. Case 1

**Figure 1:**
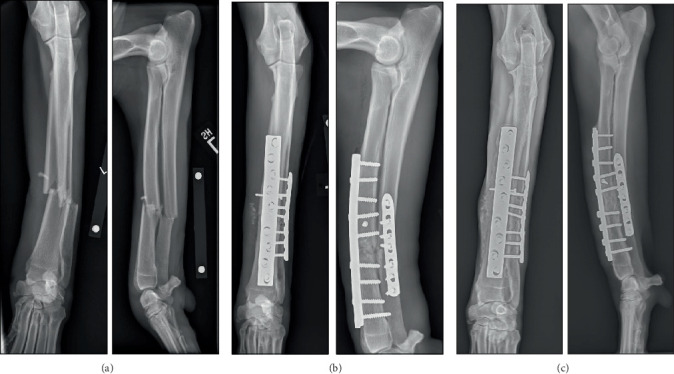
Orthogonal views of the left radius and ulna of a 6.5-year-old male Great Dane obtained under sedation at three time points. (a) Initial presentation. A closed left distal diaphyseal transverse fracture of the radius and ulna with mild comminution and a fissure on the cranial aspect of the distal portion of the radius with associated soft tissue swelling was found. (b) Immediately after surgery. Adequate fracture reduction with a 2.7mm lag screw spanning a radial fissure in the proximal fragment of the radius. A 4.5mm nine-hole broad DCP with nine bicortical screws was placed on the cranial surface of the radius as a neutralization plate with a 3.5mm seven-hole LCP on the lateral surface of the ulna with seven bicortical screws. Bone graft can be seen in the fracture site. (c) Two years after surgery. The implant positions were like earlier radiographs. Clinical union of the fracture site was achieved. A moderate periosteal reaction was present on the lateral and caudal surfaces of the radius at the level of the previous fracture with lucency surrounding the most proximal radial screws.

A 6.5-year-old male Great Dane weighing 63 kg was presented for lameness after being hit by a car. Clinical signs on admission to UW Veterinary Care included a non-weight bearing left thoracic limb lameness with moderate soft tissue swelling and instability at the level of the antebrachium. Neurologic assessment of the limb was limited due to fracture pain. On assessment, the limb had intact motor and reflexes as well as pain sensation in all digits. Proprioception could not be assessed due to nonweight-bearing lameness. Multiple superficial lacerations to the medial left antebrachium, left medial carpus, left digits, left lateral stifle, and ventral chin were also found. The patient was sedated, and all wounds were explored, cleaned, and lavaged with sterile saline. A < 1 cm puncture noted on the left antebrachium was present near the fracture site with an absence of pocketing in any direction and no communication with the fracture based on probing. An abrasion of the left medial carpus distal to the fracture site was explored and found to contain a 2-cm pocket. The remaining wounds were partial thickness. Radiographs of the left antebrachium showed a closed left distal diaphyseal transverse ulnar and radial fractures with mild comminution and associated soft tissue swelling (Figure 1). Right antebrachial radiographs were made for surgical planning, and no orthopaedic abnormalities were noted. A complete blood count and a serum biochemistry panel showed no clinically noteworthy findings.

The patient was hospitalized, stabilized, and managed overnight on 5 mg/kg of pregabalin, hydromorphone at 0.05 mg/kg, and amoxicillin/clavulanic acid at 30 mg/kg IV every 8 hours due to the presence of open wounds. ORIF was performed through a lateral surgical approach [6]. During surgery, intraoperative cefazolin was administered at 22 mg/kg every 90 min. Initially, a 2.7mm screw was placed in lag fashion from lateral to medial, proximal to the radial fracture to compress a large sagittal fissure. A sagittal fissure distal to the fracture was avoided during screw placement. A 4.5mm nine-hole broad DCP with nine bicortical screws (DePuy Synthes Vet, West Chester, Pennsylvania) was placed on the cranial surface of the radius as a neutralization plate and a 3.5mm seven-hole LCP (DePuy Synthes Vet, West Chester, Pennsylvania) on the lateral surface of the ulna and secured with seven bicortical screws. The broad 4.5mm radial plate had a bone screw density of 1, a plate span of 52%, a plate span ratio of 17.5, and a plate working length of 21.7 mm. The 3.5mm ulna LCP had a bone screw density of 1, a plate span of 28.5%, a plate span ratio of 6.5, and a plate working length of 15.6 mm. Bone screw width was ~17.3% and 23.3% of bone diameter for the 4.5mm and 3.5mm implants, respectively. Given the patient's age, a bone graft was collected routinely from the right proximal humerus and placed within the fracture sites to augment the healing response. The incision was closed in three layers; the fascial layer was closed with 3-0 polyglyconate in a continuous pattern, the subcutaneous layer was closed with 3-0 Biosyn (Covidien, Mansfield, Massachusetts) in a continuous pattern, and the skin was closed with 3-0 nylon in a simple interrupted pattern. The superficial wounds on the medial antebrachium and carpus were debrided, lavaged, and closed. Postoperative radiographs showed appropriate placement and alignment of the fractures (Figure 1). The dog was sent home on a 7-day course of 13.75 mg/kg PO BID of amoxicillin/clavulanic acid due to the presence of full thickness skin wounds.

A physical examination 2 weeks later revealed a swollen left thoracic limb and persistent Grade 4 of 5 lameness. A fluid pocket was present on the left thoracic limb at the location of a previous wound, which was sampled and submitted for culture. Recheck radiographs were declined by the owner. Treatment with amoxicillin/clavulanic acid at a dose of 13.75 mg/kg PO BID was given for a further 2 weeks. Culture of the wound confirmed a beta hemolytic *Staphylococcus* sp. infection susceptible to the prescribed antibiotics. At the next recheck examination 20 days after the initial recheck, there was still a Grade 4 of 5 left thoracic limb lameness. The infection had resolved clinically, but the antibiotic course was continued for an additional 4 weeks to minimize the risk of recurrence. The owner declined further follow-up at the appointment.

Clinical follow-up was performed 2 years after surgery. The dog had a history of persistent lameness after exercise. An intermittent recurrent abscess at the site of one of the wounds had resolved with antibiotic administration by their primary care veterinarian before presentation. On examination, a Grade 1 of 5 left thoracic limb lameness was noted. There was reduced carpal range of motion such that the carpus was mostly extended at the trot. Firm thickening of the left antebrachium and carpus was found on physical examination together with mild muscle atrophy. Range of motion in the left carpus was decreased; the contralateral carpal range of motion was within normal limits. Healing of the fractured radius and ulna was confirmed radiographically (Figure 1). Radiographically, implant position was the same as immediately after surgery. There was moderate periosteal reaction on the lateral and caudal surfaces of the radius at the level of the previous fracture, and lucency surrounding the most proximal radial screws, which was considered a consequence of cyclic mechanical loading. The owner declined further clinical follow-up.

## 3. Case 2

**Figure 2:**
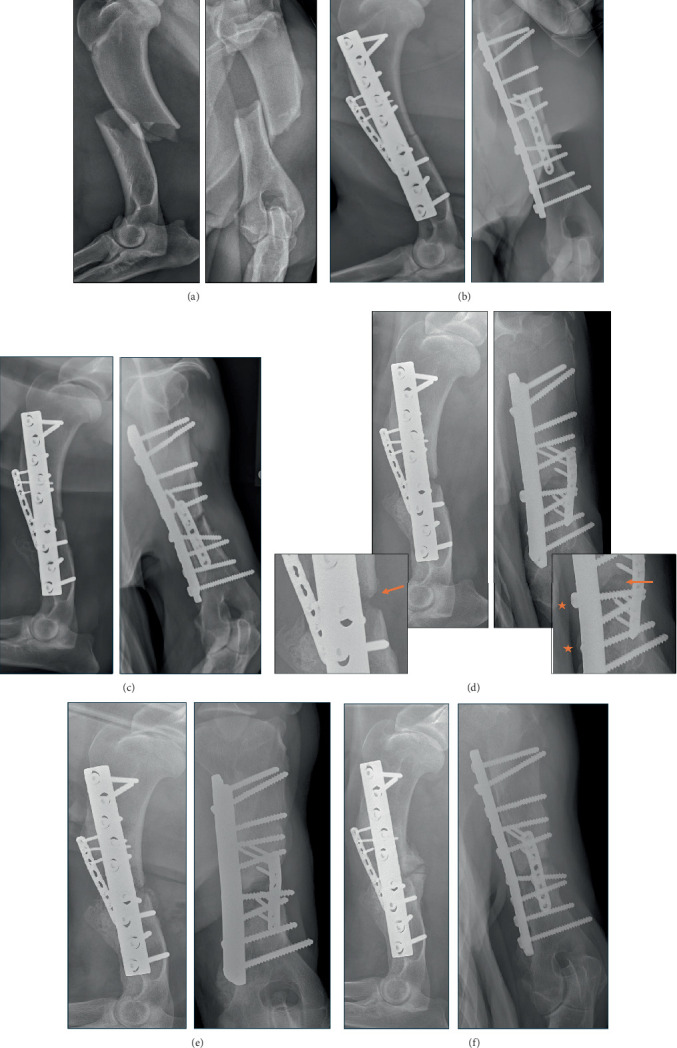
Orthogonal views of the left humerus of a 1-year-old male Saint Bernard obtained under sedation at six time points. (a) Initial presentation. A complete, closed, craniomedially displaced, short-oblique fracture of the mid diaphysis of the left humerus with associated soft tissue swelling. (b) Immediately after surgery. A 2.7mm eight-hole LCP was placed on the cranial surface of the humerus using a mix of cortical and locking screws of appropriate length. A 4.5mm broad nine-hole DCP plate was placed on the medial surface of the humerus as a neutralization plate. (c) Seven weeks after surgery. No change in the implants was found. Bone resorption was present at the fracture site with minimal callus formation. (d) Three months after surgery: Persistent resorption of bone at the fracture site. Limited new bone formation at the caudal aspect of the humerus with no apparent bridging of any cortices (arrow) indicating delayed union. Loosening of the 4.5mm screws adjacent to the fracture was found (star). (e) Five months after surgery and 5 weeks after surgical revision. Implants remain static in position. Remodeling of the bone graft and improved callus formation is present, with clinical union not yet achieved. (f) Eight months after surgery. There were no changes in the position of the implants. Clinical union of the humeral fracture has occurred with bridging of the cranial, caudal, and medial cortices.

A 1-year-old male Saint Bernard weighing 64 kg presented to an emergency clinic for an acute onset lameness, suspected to be secondary to motor vehicle trauma. On physical examination, the patient had a nonweight-bearing lameness in the left thoracic limb. The limb was unstable upon palpation at the level of the humerus. A complete, closed, craniomedially displaced, short oblique fracture in the mid diaphysis of the left humerus with associated soft tissue swelling was identified on orthogonal radiographs of the left humerus (Figure 2). Further diagnostics showed no significant abnormalities. Neurologic assessment was limited due to the nonweight-bearing nature of the lameness.

The patient was transferred to UW Veterinary Care for surgical stabilization of the left humeral fracture. The dog was managed on hydromorphone at 0.05 mg/kg, carprofen at 2.2 mg/kg, and gabapentin at 10 mg/kg before surgery. ORIF was performed through a standard medial approach to the humerus [6]. The patient received 22 mg/kg of cefazolin at induction and every 90 min throughout surgery as prophylactic treatment. Initially, a 2.7mm eight-hole LCP (DePuy Synthes Vet, West Chester, Pennsylvania) was contoured and placed on the cranial humerus to facilitate fracture reduction using both cortical and locking screws of appropriate length. This plate had a bone screw density of 0.625, plate span of 29.7%, plate span ratio of 7.2, and plate working length of 23.6mm. A 4.5mm broad nine-hole DCP plate (DePuy Synthes Vet, West Chester, Pennsylvania) was then contoured and placed on the medial humerus as a neutralization plate and was secured using appropriate cortical screw placement. This plate had a bone screw density of 1, plate span of 58.9%, plate span ratio of 14.4, and plate working length of 19 mm. Screw width was ~5.8%–9.3% and 9.2%–15.8% of bone diameter proximally and distally for the 2.7mm and 4.5mm implants, respectively. The fracture site was not grafted because of the dog's young age. The incision was closed in three layers with the muscle and fascial layers being closed with 0 or 2-0 polyglyconate in a simple continuous pattern, the subcutaneous tissue being closed in a simple continuous pattern with 2-0 Biosyn (Covidien, Mansfield, Massachusetts), and the skin being apposed with 3-0 nylon in a simple interrupted pattern. Postoperative radiographs showed acceptable anatomical reduction (Figure 2). The patient left the hospital 2 days after surgery on prophylactic oral cephalexin (22 mg/kg BID for 10 days) and trazodone (5 mg/kg) as needed to facilitate strict activity restriction with cage rest and no off-leash activity as he was normally a very active dog. At the time, he was non-weight bearing lame on the left thoracic limb.

At recheck examination 6 weeks later, the patient remained Grade 5 of 5 on non-weight bearing lame on the left thoracic limb with occasional toe-touching and severe muscle atrophy of the shoulder. The activity of the dog had not been restricted, and he had been allowed to roam off leash. No evidence of implant loosening or breakage was found on recheck radiographs of the left humerus. Resorption of bone at the fracture site was present with minimal active new bone formation (Figure 2). Activity restriction to a leash and cage rest was again recommended, and the patient was continued on oral sedatives (trazodone 5 mg/kg as needed) and nonsteroidal anti-inflammatory drug medication (carprofen 2.2 mg/kg BID).

At the recheck examination 6 weeks after his previous recheck, the patient continued to have a Grade 3 of 5 left thoracic limb weight-bearing lameness. Lameness was worse after exercise, according to the owner's history. He also had a new Grade 2 of 5 lameness in the left pelvic limb. Severe atrophy of the lateral shoulder musculature was found, with discomfort on shoulder range of motion together with decreased flexion of the left elbow. Neurologic examination was difficult to assess due to the patient's temperament. Additional radiographs of the left humerus showed delayed union fracture with loosening of screws adjacent to the fracture. Revision surgery was recommended to the owner. Under general anesthesia, a mini medial approach to the humeral fracture site was made. After fracture site debridement, autogenous cancellous bone was collected from the left proximal humerus and mixed with 3 cm^3^ of allogeneic demineralized bone matrix (Veterinary Transplant Services Inc., Kent, Washington) to increase the total volume of the graft. This was placed within the fracture site. The loose screws adjacent to the fracture were retightened to enhance compression of the plate against the bone. The incisions were closed in the same three-layer closure as his previous surgery. The dog was sent home on oral cephalexin (22 mg/kg BID for 10 days) as surgical prophylaxis. The fracture site and surrounding soft tissues were cultured. The culture from the soft tissues grew methicillin-resistant *Staphylococcus pseudintermedius*. No bacteria were isolated from the culture of the fracture site and plate. The cephalexin was consequently discontinued, and oral trimethoprim/sulfamethoxazole was given (16 mg/kg BID for 1 week). Five months after the initial surgery, another recheck examination confirmed persistent Grade 3 of 5 left thoracic limb lameness. Recheck radiographs of the left humerus showed evidence of progressive remodeling and reorganization of the bone graft and healing of the fracture (Figure 2).

The dog was again reexamined for continued Grade 3 of 5 lameness of the left thoracic limb 8 months after the initial surgery. Physical examination revealed severe muscle atrophy most prominently in the infraspinatus and extensor carpi ulnaris muscles, with moderate muscle atrophy of the remainder of the lateral musculature of the left shoulder. Neurologic examination revealed delayed proprioception of the left thoracic limb and abnormal placement of the paw while walking. Segmental reflexes could not be assessed due to the patient's temperament. Radiographs showed clinical union of the humeral fracture with progressive remodeling of the fracture site callus. On electromyography, spontaneous activity of the infraspinatus muscle innervated by the suprascapular nerve and extensor carpi ulnaris muscle innervated by the radial nerve was seen consistent with a diagnosis of a brachial plexus injury and muscle denervation. The patient's continued lameness was considered mechanical from neurological injury secondary to the initial trauma, and continued monitoring was recommended.

## 4. Discussion

The purpose of this case report was to describe the use of 4.5mm DCP plate and screw fixation for long bone fracture repair in giant breed dogs and assess outcomes, as clinical outcomes with use of these implants are poorly documented in dogs. A key surgical goal is rigid fracture fixation since inadequate fixation is the most common reason for revision surgery and plate removal in dogs [7]. Plating both the radius and ulna in Dog #1 likely also added to the stability of fracture fixation due to the shared load-bearing between both bones, decreasing the force applied to the radial plate.

In looking at the biomechanics of fracture repair, an adequate construct must be able to resist the forces applied to the limb during habitual activity during the healing period. Construct failure can occur at any point in the healing process before clinical union. In a recent study, the average time to failure of the construct was 45 days, indicating failure most often occurs in the early to mid-stage of healing [3]. In large or giant breed dogs, the construct loading is greater due to their increased size, thus increasing the risk of plate or screw failure. Bone screw breakage or loosening, plate bending, or plate breakage can occur in dogs, but screw loosening or breakage is the most common type of implant failure in plate repair of long bone fractures [3]. Implant AMI is an important consideration in the preoperative planning of a fracture repair when it comes to a construct's strength. It is known from mechanical studies of the strength of 3.5mm broad plates that the 3.5mm cortical screws tend to be a point of failure ex vivo [3, 8]. In the cases included here, 4.5mm cortical screws were used, which have an AMI of 4.0 mm^4^, which is 2.5-fold greater than their 3.5mm counterparts, which have an AMI of 1.6 mm^4^ [4]. In addition, when comparing the AMI of the 3.5mm broad plate (54.9 mm^4^) and the 4.5mm broad plates (121.5 mm^4^) used in these cases, the AMI is much greater in the 4.5mm implants.

Currently, there is minimal information on the clinical use of 4.5mm plates and screws for fracture repair in dogs. However, based on the results of a study in 1977 by Brinker et al. [5], the recommended plate size for a humeral or radial fracture in a dog weighing more than 50 pounds was a 4.5mm narrow plate, and a 4.5mm broad plate was recommended for dogs over 100 pounds with a humeral fracture [5]. Though this was examined before widespread use of the 3.5mm broad and locking plates, it demonstrates the continued relevance of the 4.5mm plates and screws when treating long bone fractures in giant breed dogs. The 4.5mm screws were 9%–17% of bone diameter in the dogs of this report, suggesting appropriate sizing. More recently, mechanical differences between the 4.5mm standard and the 3.5mm broad plates, which have similar dimensions, have been studied [2, 8]. Although the 3.5mm broad plate constructs can use more bone screws per segment compared to a the 4.5mm plate, which is helpful in canine bone with relatively thin cortices, constructs with fewer 4.5mm screws have similar mechanical properties [8]. During the typical cyclic fatigue failure of fracture constructs that develops clinically in patients with mechanical fracture construct complications, the stronger 4.5mm bone screws are likely advantageous. This is consistent with a similar study looking at various implant types that measured their resistance to bending and torsion [9]. This paper also found that the 4.5mm broad DCP construct was nearly twice as stiff and, therefore, more resistant to bending and torsion than the 3.5mm DCP broad plate [9]. Although these studies were all performed ex vivo, they offer insight into why implant failure was not encountered even in the face of delayed healing associated with the major complication of surgical site infection in the first case [10] and atypical partial brachial plexus injury from the original trauma in the second case.

In Case 1, we do not know whether delayed union was present as detailed follow-up was declined during the healing period. However, recurrent surgical site infection is associated with delayed union in dogs [11, 12]. In Case 2, fracture healing was substantially delayed in a young patient where rapid fracture healing would be expected. Delayed healing could have been secondary to poor activity restriction, the lack of bone grafting in the initial fracture repair even though the patient was of a young age, or from stress shielding from use of a stiff construct, or neuronal injury. Based on bone density measurements in dogs treated with 4.5mm leg lengthening plates, which are particularly stiff, there is no evidence that the 4.5mm nonlocking implants are particularly associated with stress shielding in dogs [13], likely because of screw loosening over time [14]. Therefore, given the loosened screws found during revision surgery in Case 2, stress shielding is unlikely to have contributed to the delayed union. Neuronal injury to the brachial plexus in Case 2, including injury to suprascapular and radial nerves or nerve roots, could have contributed to delayed fracture healing as the nervous system is known to have regulatory effects on the bone modeling responses to mechanical loading based on mechanistic studies in a rat model [15]. The pattern of neurologic deficits suggests that nerve injury occurred at the time of the trauma and not secondary to surgical complications, as the plate was placed on the medial aspect of the humerus. This is atypical, as most brachial plexus injuries result in severe lameness and only ~10% are affected with weight-bearing monoparesis [16]. Although screw loosening occurred in this second case, likely because of the development of fracture site micromotion over time from poor activity restriction and delayed healing, breakage of the 4.5mm screws did not occur. The interface between the screw threads and the bone remained intact as tightening of the screws to a typical final torque was possible during the revision surgery. An association between implant failure and delayed union has been established, but which typically develops first is often unclear [11]. Delayed union has been associated with the development of implant failure, with complications associated with the screws being the most common problem via either breakage/bending or loosening [3].

## 5. Conclusion

The use of 4.5mm DCP implants for long bone fracture repair in two giant breed dogs promoted successful fracture healing and an acceptable clinical outcome in the face of major complications associated with surgical site infection and delayed bone healing, which were considered major complications [10]. Implant breakage did not occur in either case, and clinical union of both long bone fractures was achieved after treatment. The use of 4.5mm plates is an important option when considering methods of fracture repairs in giant breed dogs, principally because of the high AMI of the 4.5mm cortical screws.

## Data Availability

Data sharing not applicable to this article, as no datasets were generated or analyzed during the current study. The clinical data used to support the findings of this study are included within the article.
